# Chitotriosidase Activity and Gene Polymorphism in Iranian Patients with Gaucher Disease and Sibling Carriers 

**Published:** 2016

**Authors:** Hadi MOZAFARI, Mohammad TAGHIKHANI, Shohreh KHATAMI, Mohammad Reza ALAEI, Asad VAISI-RAYGANI, Zohreh RAHIMI

**Affiliations:** 1 Department of Clinical Biochemistry, Faculty of Medical Sciences, Tarbiat Modares University, Tehran, Iran; 2 Department of Biochemistry, Pasteur Institute of Iran, Tehran, Iran; 3 Department of Pediatric, Faculty of Medicine, Shahid Beheshti University of Medical Sciences, Tehran, Iran; 4 Department of Clinical Biochemistry, Medical School, Kermanshah University of Medical Sciences, Kermanshah, Iran

**Keywords:** Gaucher Disease, Chitotriosidase, 24 bp Duplication, Polymorphism, PCR-RFLP

## Abstract

**Objective:**

Chitotriosidase (CT) activity is a useful biomarker for diagnosis and monitoring of Gaucher disease (GD). Its application is limited by some variants in the CT gene. Two main polymorphisms are 24 bp duplication and G102S led to reduce CT activity. The aim of this study was to determine these variants influencing on plasma CT activity.

**Materials & Methods:**

Blood samples were collected from 33 patients with GD, 15 sibling carriers and 105 healthy individuals serving as controls. CT activity was measured using 4-methylumbelliferyl-β-D-N,N′,N″triacetylchitotrioside substrate in plasma samples. The CT genotypes of 24 bp duplication and G102S variants were determined using PCR and PCR-RFLP.

**Results:**

Untreated GD patients had a significantly higher CT activity compared to treated patients (P = 0.021). In addition, chitotriosidase activity in carriers was higher rather than controls. Allele frequencies of 24 bp duplication in GD patients, sibling carriers and controls were 0.21, 0.266 and 0.29 and for G102S were 0.318, 0.366 and 0.219, respectively. Different G102S genotypes had not significant effect on CT activity. Chitotriosidase activity has a positive correlation with age in normal group, carriers, and negative correlation with hemoglobin in GD patients. Using cut-off level of 80.75 nmol/ml/h, sensitivity and specificity of CT activity were 93.9% and 100%, respectively.

**Conclusion:**

Chitotriosidase activity is a suitable biomarker for diagnosis and monitoring of GD. Determination of 24 bp duplication is helpful for more accurate monitoring the GD patient’s therapy. However, it seems that, specifying of the G102S polymorphism is not required for Iranian GD patients.

## Introduction

Gaucher Disease (GD) is an inherited metabolic disorder of lysosomal storage disease (LSDs) occurred by deficient activity of the glucocerebrosidase (GBA) (1). According to central nervous system symptoms, GD is classified into three main types including: Type I of the GD (OMIM 230800) or non-neuronopathic form associated with organomegaly, anemia and thrombocytopenia, Type II (OMIM 230900) or acute neuronopathic disease and Type III (OMIM 231000) of the GD or chronic neuronopathic form of the disease manifested: seizure, supranuclear horizontal gaze palsy or other eye symptoms, and mental retardation. 

Generally, GD is diagnosed by measurement of β-glucosidase activity in leukocytes and fibroblasts or by genetic testing of GBA gene as confirmatory test (1-3).

In addition, several serum analytes have been described for monitoring GD including pulmonary and activationregulated chemokine (PARC/CCL18), angiotensinconverting enzyme (ACE), macrophage inflammatory proteins (MIP)-1α, MIP-1β, CD163 and etc. (1, 4-6). 

However, these are moderately increased but, nonspecific to this disease (5-6). Now, the most powerful biomarker for monitoring of GD is chitotriosidase (CT; EC 3.2.1.14) (5-7), an enzyme secreted in plasma by activated macrophages from different tissues (7). Its activity is highest in untreated Type 1 GD patients, which is averagely 600-fold greater than that in controls and correlated with disease severity. Note that, plasma CT levels gradually decreased during ERT (enzyme replacement therapy). Therefore, it is useful for monitoring disease severity and the effectiveness of therapy in GD (6).

Besides, CT is useful on the clinical management of GM1-gangliosidosis (8), Alzheimer, amyotrophic lateral sclerosis, atherosclerosis, β-thalassemia and malaria (9-11).

The CT gene (MIM 600031) is located on the chromosome 1q31-q32 and consists of 12 exons (12). 

A recessive inherited mutation of CT gene, which consists of c.1049_1072dup24 or 24bp duplication (Dup24) in exon 10, encodes inactive CT protein lacks the 29 amino acids and results in an inactive enzyme (3). About 6% of Caucasians are homozygous and 30% to 40% are heterozygous for the Dup24 null allele (6). Another common polymorphism, p.G102S (Glycine 102 Serine), interferes with CT catalytic properties when using 4-methylumbelliferyl-β-D-N, N′, N″triacetylchitotrioside (4MU-chitotrioside), causing activity reduction of CT levels and consequent misinterpretation (5). Thus, determination of GD patient CT genotypes is important to correlate the plasma CT activity with disease severity for therapeutic monitoring. 

The aim of present study was to report the CT activity and frequency of the Dup 24 and G102S missense mutation in Iranians GD patients, obligate carriers and normal population. Especially, this analyte changes in response to treatment and would be useful to individualize dose.

## Materials & Methods

**Samples**

We conducted our study on 33 GD patients and 15 sibling carriers approved by PCR-RFLP or sequencing method. Four of patients had GD type III and referred to Mofid Children’s Hospital and Ali-Asghar Children’s Hospital of Tehran, Iran. All patients were previously diagnosed with GD according to fluorometric assay of the GBA activity, having the gaucher cells in the bone marrow or GBA gene analysis (unpublished data). 

Twenty-one of them received Cerezyme therapy as average intravenous injection dose: 30-60 U/kg per 2 weeks. The average time of ERT was 4.05 ± 1.87 yr. 

Moreover, we selected an age and sex matched group of 105 normal individuals as controls.

Informed written consent was obtained from individuals or their parents before participation. This study was approved by Ethics Committee of Tarbiat Modares University and Pasteur Institute of Iran.

The characteristics of studied subjects are shown in Table 1. Three ml peripheral blood was collected from each subject, mixed with EDTA, and then 65 μl aliquots were spotted on Whatman 903 filter paper and dried at room temperature. Finally, blood spot, plasma and cell pellets kept at −80 °C until time of analysis. In addition, cell blood count (CBC) was measured by a hematology analyzer (Sysmex, Japan). 

**Fluorimetry Assays**

GBA activity was determined on dry blood spot sample by a modified method (13). Chitotriosidase in plasma was measured based on the method of Hollak et al. (14). Briefly, 5 μl of plasma (in the case of GD, 1 to 50 diluted with water) was mixed with 100 μL of 0.022 mmol/L 4MU-chitotrioside (Sigma-Aldrich Co, USA) in McIlvain’s buffer (100mmol-1 citric acid and 200 mmol-1 sodium phosphate, pH 5.2) and incubated at 37 °C for 30 min at dark place. The reaction was stopped with 1395μl of 0.2 M Na2CO3/glycine pH 10.5. Fluorescence activity was measured by RF-5000, Shimadzu, Japan (Excitation 366 nm, Emission 448 nm).

**Table1:** Characteristics of the study population

**Parameter**	**GD Patients (n=33)**	**Controls (n=105)**	**Sibling Carriers (n=15)**	**P value**
**Sex (male%)**	14 (42.4%)	51 (48.6%)	9 (60%)	0.082
**Age (years)**	11.5 ± 8.1	9.9 ± 6.6	14.33 ± 8.6	>0.05
**GBA (μmol/lit/h)**	0.63 ± 0.38	3.47 ± 0.90	2.15 ± 0.44	<0.001
**CT (nmol/ml/h)**	9061 ± 11031	15.7 ± 11.5	25.8 ± 14.9	<0.001
**WBC (×103/μl)**	6.07 ± 1.96	6.99 ± 1.63	6.96 ± 2.32	0.014
**RBC (×106/μl)**	4.51 ± 0.70	5.09 ± 0.41	5.16 ± 1.01	<0.001
**Hemoglobin (g/dl)**	12.17 ± 2.29	14.05 ± 1.41	15.09 ± 3.31	<0.001
**Platelet (×103/μl)**	154.03 ± 63.09	273.22 ± 69.12	245.33 ± 68.4	<0.001

Enzyme activities were calculated based on a calibration curve of 4-methylumbelliferone (4-MU) for each assay.

**CT genotyping**

DNA was extracted by phenol-chloroform method (15). Briefly, the PCR amplification of both targets was carried out using previously described protocols (16, 17). For 24 bp duplication genotyping (16), products separated on 2.5% agarose gel. The presence of duplication in both alleles (Dup24/Dup24; Homozygous) genotype produces only a 99bp fragment, wild genotype (Wt/Wt), produced a 75bp fragment. The presence of both 99 and 75bp fragments showed Wt/ Dup24 genotype (Heterozygote). The G102S mutation genotyping was performed by PCR product (259bp) digestion with HpaII restriction enzyme (Thermo Scientific™). Digestion yielded three bands including 259 or 240 and 19bp (17). In the presence of mutation, 259bp band remain intact. The wild type allele renders two bands of 240 and 19bp.

Finally, digested products were separated on 3.5% agarose gel.

**Statistical Analysis**

Data were analyzed using SPSS 16.0 (Chicago, IL, USA) and GraphPad Prism 6 software. The normality of variables was tested by Kolmogrov-Smirnov test. Continuous data were analyzed by Independent t-test or Mann–Whitney test, and Kruskal-wallis or ANOVA test. To evaluate the degree of linear association between different variables, Pearson or spearman correlation coefficients were calculated. The genotypes and allele frequencies of Dup24 and G102S variations between groups were compared using the Chi-square analysis. 

The level of significance was set at P<0.05. Finally, the clinical performances of CT activity for GD measured using receiver operating characteristic (ROC) curves. Cut-off value that provided the best combination of sensitivity and specificity was determined. 

## Results

Characteristics and the laboratory data of three main groups are presented in the Table 1. All groups in terms of age and sex were matched. As expected, significant differences in plasma CT activity values were found in patients with GD (9061 ± 11031) compared to the normal subjects (15.7 ± 11.5, P<0.001) or sibling carriers (25.8 ± 14.9, P<0.001). Particularly, GD treated with ERT (5827 ± 8288) had significant lower levels of CT activity than those were untreated (14719 ± 13199, P=0.021), and patients with type III of disease insignificantly had a higher CT activity (14773 ± 16108) than those with GD type I (8273 ± 10294). 

According to Table 2, absence of CT activity was detected in two out of the 33 GD patients (6.1%), one out of carriers (7.7%), and 11 out of 105 control individuals (10.5%). These individuals were excluded from the statistical analysis concerning plasma CT activity and correlations. The Dup 24 genotypes distribution of GD patients, sibling carriers and controls are stated in Table 2. All patients, carriers and controls with no detectable CT activity were homozygous for the 24bp variation. 

**Fig 1:**
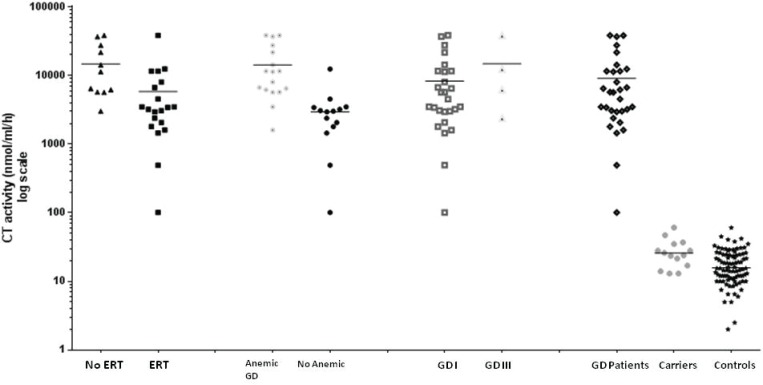
Chitotriosidase activity for GD patients and controls according to ERT, GD type, Dup24 and G102S genotypes

CT activity in three main groups and sub-groups are shown in Figure 1. 

The G102S genotypes distribution of GD patients, sibling carriers and controls are stated in Table 2. 

**Table 2:** Distribution of Dup24 and G102S genotypes, alleles, and haplotypes in GD patients, controls and sibling carriers

**CT Genotypes**	**GD Patient** **number (%)**	**CT Activity**	**Control** **number (%)**	**CT Activity**	**Carriers** **number (%)**	**CT Activity**
**24 Duplication**						
**A- Wt/Wt (Wild)**	21 (63.6)	10593 ± 11389	55 (52.4)	22.2 ± 10.7^a^	8 (53.3)	34.06 ± 14.4^a^
**B- Wt/24Dup (Hetero)**	10 (30.3)	7654 ± 10886	39 (37.1)	11.2 ± 6.9	6 (40)	19.25 ± 6.67
**C- 24Dup/24Dup** **(Homo)**	2 (6.1)	0	11 (10.5)	0	1 (6.7)	0
**24Dup allele (%)**	14 (21.2)	-	61 (29.04)	-	8 (26.6)	-
**G102S genotypes**						
**D- GG (Wild**)	17 (51.5)	6935 ± 9585	64 (61)	16.3 ± 11.4	7 (46.6)	28 ± 11.4
**E- GA (Hetero)**	11 (33.3)	11589 ± 3490	36 (34.3)	14.7 ± 11.9	6 (40)	23.8 ± 20.3
**F- AA (Homo)**	5 (15.2)	10723 ± 15190	5 (4.8)	15.9 ± 11.2	2 (13.4)	24.5 ± 14.8
**G- GA+AA**	16 (48.5)	11318 ± 12290	41 (39.04)	14.9 ± 11.7	9 (60)	24 ± 18.1
**A allele (%)**	21 (31.8)	-	46 (21.9)	-	10 (33.3)	-
**Haplotypes** **H- Both Wild (Ref)**	8 (24.2)	10610 ± 13110	32 (30.5)	23.6 ± 9.4	5 (33.3)	31 ± 11.4
**I- 24Dup/Wt – GG**	7 (21.2)	4717 ± 2163	23 (21.9)	12.3 ± 2.9^b^	2 (13.3)	20.5 ± 10.6
**J- Wt/Wt – GA**	8 (24.2)	10494 ± 8175	20 (19)	20.02 ± 12.8	2 (13.3)	41.2 ± 27.9
**K- 24Dup/Wt – GA**	3 (9.1)	14509 ± 20459	14 (13.3)	9.6 ± 6.6^c^	4 (26.6)	20.1 ± 6.2
**L- Wt/Wt – AA**	5 (15.1)	10723 ± 15190	3 (2.8)	20.3 ± 9.7	1 (6.7)	35
**M- 24Dup/Wt – AA**	0 (0)	-	2 (1.9)	9.2 ± 13.1	0 (0)	-

compared to H (reference group), in controls (p<0.001). c; Statistically significant to H, in control group (p<0.001).

a ; Statistically significant comparison between row A and B in controls (p<0.001) and in carriers (p=0.039)

b ; Statistically significant

Furthermore, untreated patients with Wt/Wt genotype (n=7, 22255 ± 4715) had a higher CT activity than those with Wt/Dup24 genotype (n=4, 5212 ± 1506, P=0.014). 

Yet, reduced CT activities in G102S haplotypes containing mutant alleles were inconsistent and nonsignificant (Table 2). CT activity in control group, had a significant positive correlation with age (r=0.314, P<0.01). On the other hand, in GD patients negative correlation between CT activity and RBC number (r=-0.382, P<0.05) and hemoglobin concentration (r=- 0.580, P<0.001) was found. Moreover, in the anemic GD patients (n=19), CT activity was significantly higher (14141 ± 13109) than those without anemia (3662 ± 3994; P<0.01; Figure 1).

The area under ROC curve (AUC) was 0.943 for CT activity (Standard Error: 0.039). Diagnostic cut-off levels with the optimum sensitivity and specificity calculated from the ROC curve was CT >80.75 nmol/ ml/h. Using this optimum cut-off level, CT had 93.9% sensitivity, and 100% specificity. Likewise, positive predictive values (PPV), negative predictive value (NPV) were 100% and 98.03%, respectively.

**Fig 2:**
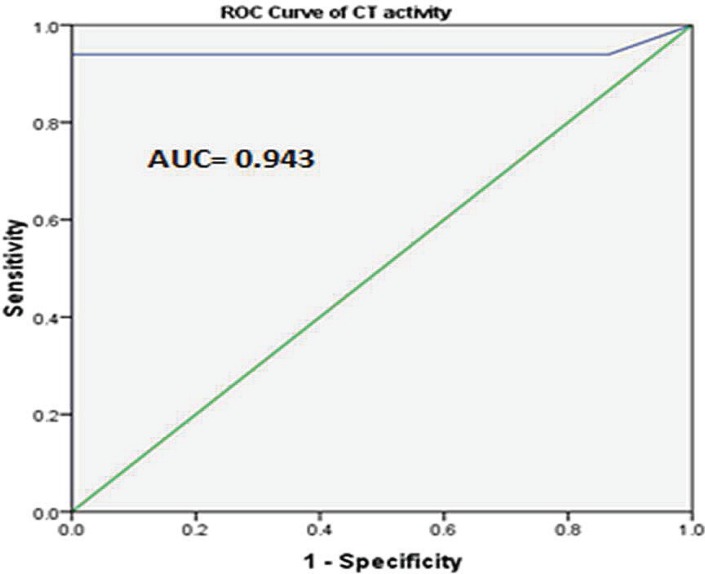
ROC curve for CT activity (AUC= area under curve

## Discussion

This is the first study conducted on an Iranian population, which describes the impact of Dup24 and G102S in the CT gene on GD patients, sibling carriers and normal population. We found that CT activity in untreated GD patients is significantly higher than normal population in accordance with previous studies (4-8). In our study, CT activity of treated and untreated patients compared to controls was about 371 and 937 times higher, respectively. Nevertheless, two studies in Brazil reported increasing of about 176 (treated GD) and 600 (untreated GD) fold CT levels (8, 18). On the other hand, it is possible that, a higher CT level in our patients is due to ethnicity difference. As well, there is a report that shows average close to a thousand-fold CT elevated in symptomatic untreated non-neuronopathic GD patients (19). According to International Collaborative Gaucher Group (ICGG) and different studies recommendation, plasma CT activity declined during ERT and could be serve as indirect measurement of GD severity (6, 20, 21). Hence, specifying the CT gene mutations that affects plasma enzyme activity is helpful for use to assess GD patients.

The allele frequencies of Dup 24 in our GD patients, carriers and controls were 0.21, 0.26 and 0.29, respectively. The allele frequency of Dup 24 was reported between zero (22) in South Africa to more than 0.50 in Korea, China and Japan normal population (23, 24). It has also been reported an allele frequency 0.22, 0.43, 0.20 in Spanish, Brazilian, and Dutch GD patients, respectively (5, 18, 25). 

We determined the G102S allele frequencies in GD patients, carriers and controls as 0.32, 0.33 and 0.22, respectively (Table 2). In different populations, allele frequencies were 0.24, 0.26, and 0.27 in Asian, African, and European normal subjects, respectively (23). 

Similarly, G102S allele in GD patients was common as 0.27, 0.31, and 0.24 in Spanish, Ashkenazi Jewish and Dutch ethnicity, respectively (5, 6, 25).

Interestingly, in untreated GD patients, CT activity in wild (Wt/Wt) genotypes was about 4-fold higher than those with heterozygote genotype. By contrast, this ratio among our controls and carriers, similar to different studies is about 2-fold (18, 26). Probably, the higher ratio observed, could be attributed to the small sample size (n=12) and selection bias, as there were eight related individuals among our GD patients. As well as, among different haplotypes of controls and sibling carriers, three sub-groups (I, K, and M rows of controls in Table 2) who had at least an allele of Dup24 had a significant lower CT activity than reference group (row H in table 2).

Remarkably, our results showed that CT levels were not different in three genotypes of G102S variation or haplotypes contain mutant allele in patients, carriers or controls, and when CT activity were analyzed in treated or untreated GD patients, separately. Our finding is in line with results of Lee et al. (23) who examined the G102S allele frequency of subjects of European, Asian and African ancestry compared to enzyme activity. This finding differs from an investigation (6), which demonstrated that using 4-MU-chitotrioside as substrate, in vitro expression of the G102S alleles had 23% of wild-type CT catalytic efficiency. As well, in another study (25), at a non-saturating concentration of 4-MU–chitotrioside, the catalytic efficiency of recombinant Serine102 CT was 70% that of wild-type Glycine102 measured. 

Like to another study (27), we found a significant elevated CT activity in sibling carriers rather than controls (P=0.007). Possibly, in carrier’s subjects a small macrophage activation enhanced CT production. 

Besides, GD carriers had lower HDL- cholesterol compared to health individuals (28). Interestingly, hypoalphalipoproteinemia is due to enhanced activation of macrophages (29).

In our study, after adjusting the CT Dup 24 genotype, GD type III patients had an insignificantly higher mean of CT activity than GD type I patients. Like us, Ries et al. (30), reported that CT levels was higher in GD type III than in GD type I.

We found a positive correlation of CT activity and age both in controls (r=0.314, P<0.01) and carriers (r=0.479, P=0.08). Consistent to our result, Kurt et al. (31) revealed that, elder persons had a higher CT activity than youths. This phenomenon could be explained by the beginning of macrophage lipid accumulation and chronic inflammation during the progression of atherosclerosis in relation to age (32).

Moreover, this correlation could be related to the relation between BMI (Body Mass Index) and plasma CT activity in children’s (27). Concordant increasing of BMI with age in health children, promotes more CT production in body. 

We indicated that, in anemic GD patients, CT activity was significantly higher than non-anemic (P<0.01).

Additionally, the CT levels in patients inversely correlated with hemoglobin concentrations (r=-0.580, P<0.001). Previously, same result was found in Dutch (19) GD patients (r=−0.304, P=0.006). Because of this fact, CT level does not indicate any particular symptom of GD and reflects the total secreted enzyme by Gaucher cells in various tissues (26). Likely, GD body burden and accumulation of glycolipid-laden macrophages (Gaucher cells) in bone marrow led to insufficient hematopoiesis and inflammation that promotes CT production. Indirectly, higher CT levels in GD patients, represents hematopoiesis deficiency. 

Different studies have reported various disease specific cut-off values for chitotriosidase based on normal populations. Based on our data, in GD group, a plasma cut-off limit of 80.75 nmol/ml/h offered 93.9% sensitivity and 100% specificity. Our control CT activity (range: 2–61 nmol/ml/h) was similar to Hollak et a.l (14) results (range: 4–76 nmol/ml/h) and also with results of Dodelson de Kremer et al. (range: 6-60.4nmol/ml/h) (33). The cut-off value with 100% of sensitivity and specificity in two studies in Brazil were 132 and 190.3 nmol/ml/h, respectively (34). 

Meanwhile, in another study (30), for 200 nmol/ml/h as cut-off, sensitivity and specificity for GD diagnosis from miscellaneous diseases were 96% and 100%, respectively. These differences emphasize the need for determination reference values in each population. 

**In conclusion, **chitotriosidase activity serves as an important marker in the diagnosis and follow-up of Gaucher disease patients. Besides, knowledge of the Dup24 genotypes and no G102S, provides optimal interpretation of the plasma CT activity for disease severity and assessing of ERT response. 
